# Synthesis, Structure, and Properties of Reduced Graphite Oxide Modified with Zirconium Phthalocyanine as a Catalyst for Photooxidation and Dye Photodegradation

**DOI:** 10.3390/molecules30214242

**Published:** 2025-10-31

**Authors:** Yuriy Gerasymchuk, Anna Wędzyńska, Damian Szymański, Maciej Ptak, Viktor Chernii, Irena Tretyakova, Anna Lukowiak

**Affiliations:** 1 Institute of Low Temperature and Structure Research, Polish Academy of Sciences, ul. Okolna 2, 50422 Wroclaw, Poland; a.wedzynska@intibs.pl (A.W.); d.szymanski@intibs.pl (D.S.); m.ptak@intibs.pl (M.P.); 2 V.I. Vernadsky Institute of General and Inorganic Chemistry, National Academy of Sciences of Ukraine, 32/34 Academik Palladin Ave., 03142 Kyiv, Ukraine; v.chernii@gmail.com (V.C.); irena.tretyakova@gmail.com (I.T.)

**Keywords:** reduced graphite oxide, zirconium phthalocyanine, composite material, ROS generation, surface area, dyes photodegradation

## Abstract

In the aspect of water purification, a photoactive hybrid material based on reduced graphite oxide (RGO) with covalently, coordinatively, and through van der Waals interactions bonded zirconium(IV) phthalocyanine (PcZr) is proposed. In the material, the phthalocyanine complex plays the role of photosensitizer, while RGO is considered a carrier, ensuring high surface area and supporting PcZr activation. The central metal atom of PcZr directly interacts with lateral active oxygen-containing surface groups of graphite oxide, mainly –OH and –COOH. Thus, the proposed method of synthesis under solvothermal conditions allowed obtaining a relatively high concentration of the dye (0.2 wt.%) in the system based on a partially reduced and exfoliated graphite oxide. Optical studies confirmed the presence of PcZr through absorption and luminescence spectra. Additionally, effective generation of reactive oxygen species was demonstrated by testing the transformation of a dye indicator (diphenylisobenzofuran). Photocatalytic activity of the system was confirmed by photooxidizing selected organic dyes (methylene blue, Rhodamine B, Brilliant Green, and Eriochrome Black T) in a water medium, tested in slightly acidic conditions under red light. The greatest overall decrease in absorption during the photodegradation test was observed for Brilliant Green, reaching 88% after 3 h of irradiation.

## 1. Introduction

Currently, many research groups are working on fabricating and examining the properties of composite materials based on various “graphene products”. Researchers are studying, among others, graphite oxide (GO) and its reduced form (RGO), graphene, expandable graphite flakes, graphene foams, carbon dots, carbon nanotubes, and fullerenes. These structures can all be functionalized with phthalocyanine (Pc) molecules linked by various types of bonds [1,2,3,4,5,6,7]. The interest stems from the wide range of applications of Pc-activated materials in science and technology, for example, in catalysis [8,9,10,11], optical limiting [3], electrochemistry [5], and chemical or biological sensing [4,12,13].

Graphene-based catalysts may play a significant role in water purification due to their useful properties, such as large surface area, chemical stability, and the ability to be functionalized [14,15,16]. In addition to the photocatalytic effect, their functions include adsorption, membrane filtration, and electrochemical purification. Thus, they can effectively remove various pollutants, including organic dyes and pharmaceuticals, from water sources [17,18,19,20]. Such kind of activity is important because industrial dyes and pharmaceuticals in waters pose significant risks to the environment and human health and are a global problem requiring urgent attention [21,22].

Various metal phthalocyanines are used in photocatalysis of organic dyes because they are excellent photosensitizers—they can absorb visible light, transferring energy to oxygen and generating reactive oxygen species (ROS), which then degrade the dyes. In the numerous works devoted to graphene-based materials with metal Pcs [23,24,25,26], the dye attachment is usually ensured by a covalent bond via lateral substituents to the phthalocyanine ring [1,2,3,4]. In our previous work on hybrid PcZr-RGO materials (where PcZr is zirconium(IV) phthalocyanine), a bridging link (an extraplanar ligand with a different chemical structure) was also used between the metal atom and the oxygen groups on the RGO surface [27,28,29,30,31]. Furthermore, the central Zr(IV) ion facilitates direct axial coordination with the oxygen groups, resulting in efficient bonding and high dye loading on carbon structures [32]. Choosing the zirconium ion for such direct coordination is not accidental. In phthalocyanine complexes, the coordination number of Zr and Hf is typically 6. After replacing the simple axial ligand (Cl^−^) with carboxyl and hydroxyl groups of GO, the coordination number of the metal increases to 8 [31]. This significantly increases the possibility of forming both covalent and coordinate bonds, as the Zr in the phthalocyanine macrocycle is covalently bonded to two nitrogen atoms and coordinated to two additional nitrogen atoms. Furthermore, upon reaction with the carrier (GO), two additional covalent and two coordinate bonds can be formed. Regarding Pc complexes with metals from the same group of the periodic table, titanium phthalocyanine is insoluble in water and organic solvents. Hafnium, on the other hand, has a significantly smaller atomic radius due to the contraction because it is located after all the lanthanides in the periodic table, and is therefore more tightly bound to the phthalocyanine ring. This reduces both the solubility of hafnium complexes and their absorption and emission coefficients. Ultimately, the widely used PcZn with 4-fold coordination only weakly accepts axial ligands and does not bind to GO [32].

As the approach that utilizes the metal’s coordination capabilities is rarely employed, this study aimed to bond the central Zr atom of the phthalocyanine directly to the oxygen groups on the surface or edges of GO. This should result in a higher concentration of Pc in the material than in similar systems where different immobilization methods were used.

The structure, morphology, and spectroscopic properties of the obtained hybrid material were investigated. The system was tested for the photooxidation of selected organic dyes. The model dyes chosen for this study—methylene blue [33,34], Rhodamine B [35,36], Brilliant Green [37], and Eriochrome Black T [38]—have been widely used to investigate the properties of photocatalysts, including graphene-based materials functionalized with phthalocyanines. Notably, all of these dyes are used in medical practice, either in the composition of antiseptic and disinfectant preparations or in medical analytics. Furthermore, Gamelas et al. [39] demonstrated that there is a strong correlation between the photodegradation of various pharmaceuticals using materials based on Pc complexes and the photodegradation of model dyes. Consequently, the material can be evaluated for its ability to absorb and photodegrade various organic pollutants from water, including pharmaceuticals.

## 2. Results

### 2.1. Fabrication, Morphology, and Composition of PcZr-RGO

**Figure 1:**
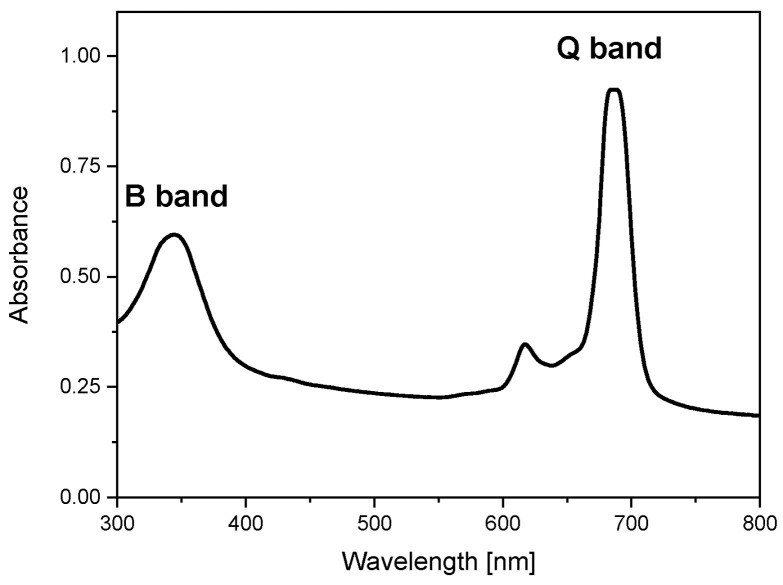
Absorption spectrum of PcZr-RGO in DMSO (Cm = 2 × 10^−5^ mol/L).

The chosen synthesis method to activate graphite oxide with Pc complex was based on the solvothermal reaction of PcZrCl_2_ with graphite oxide in the presence of N,N′-dimethyl sulfoxide (DMSO) as a solvent. The presence of PcZr in the system was initially confirmed by the UV-Vis absorption spectrum of the sample dispersed in DMSO (Figure 1). The indicator of the effectiveness of the reaction was the presence of two characteristic phthalocyanine bands, B and Q, well seen in the UV and red regions (343 nm and 685 nm), respectively. Phthalocyanines generally have a very high molar absorption coefficient (10^4^–10^6^ dm^3^·mol^−1^·cm^−1^); thus, their bands are clearly visible in the material despite the relatively low PcZr content in the carbon carrier.

To compare synthesis efficiency, another reaction was performed under the same conditions, where 1,2,4-trichlorobenzene (TCB) was used instead of DMSO. The choice of solvent for the solvothermal reaction depends on the type of active groups present on the GO surface. In general, DMSO is mainly used for ligands (or other molecules) bound with the metal through –OH and –COOH groups [40]. Meanwhile, TCB is added for complexation with the C=O groups (β-diketones), and theoretically, with epoxy groups as well [41]. In current studies, the reaction between PcZrCl_2_ and GO was much harder and less effective in trichlorobenzene. Consequently, the PcZr absorption bands in this material were much weaker compared to those in the DMSO-derived sample (Figure S1 in the Supplementary Materials).

**Figure 2:**
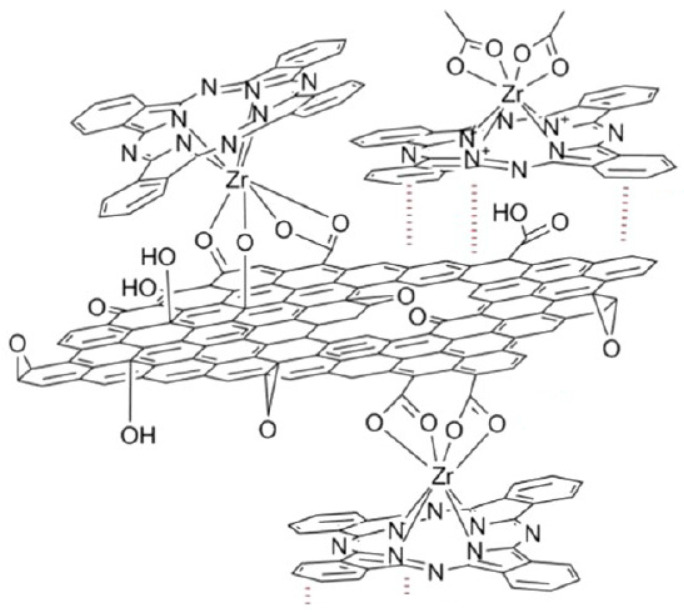
Scheme of possible binding of PcZr with RGO based on [32].

As noted in the work of Jurow et al. [32], PcZr can bind to RGO sheets via covalent and coordinate bonds, as well as interact directly via van der Waals bonds (Figure 2).

The mid- and far-infrared spectroscopic measurement (FTIR) data are presented below:

PcZrCl_2_: 1605 (w), 1500 (m), 1465 (w), 1415 (m), 1385 (w), 1330 (s), 1310 (w), ν (C-O) 1285 (s), 1155 (m), 1115 (s), 1070 (s), 1050 (s), 950 (w), 890 (s), 870 (w), 825 (m), 790 (m), 765 (m), 745 (s), 730 (s), 630 (w), 565 (w), 500 (m), 430 (m), 345 (m) [m_asym_(Zr-Cl)], 315 (s) [m_sym_(Zr-Cl)]. GO: υ (C=O) 1720–1700 cm^−1^, υ (C-O) 1220–1200 cm^−1^, υ (O-H) 3600–3550 cm^−1^.

For the PcZr-RGO, a disappearance of signals attributed to the different types of Zr-Cl bond vibrations was observed, and the appearance of corresponding signals of Zr-O bond vibration in the range of 750–800 cm^−1^ [40]. Most of the signals corresponding to the vibration bonds in the phthalocyanine macrocycle were also observed in the material, but with much lower intensity due to the very low concentration of PcZr.

**Figure 3:**
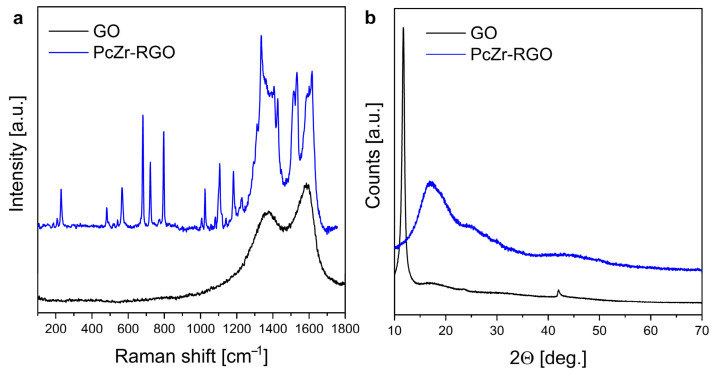
Comparison of Raman spectra (**a**) and diffractograms (**b**) of GO and PcZr-RGO.

The Raman spectra of the obtained material and the GO precursor are presented in Figure 3a. They confirmed the presence of both graphite oxide and Pc rings in the prepared system. Two broad modes seen around 1350 cm^−1^ and 1580 cm^−1^ are characteristic of different allotropes of carbon. The D mode arises from a breathing mode of κ-point phonons of A_1g_ symmetry (ca. 1350 cm^−1^). The G band (at 1580 cm^−1^) is associated with the doubly degenerated E_2g_ phonon mode at the Brillouin zone center. It arises due to the in-plane vibration of the sp^2^ carbon atoms and is typical in graphitic materials and other sp^2^ carbon systems [42,43]. Other bands seen in the spectrum correspond to the vibrations of PcZr (as confirmed also by the FTIR measurement).

The X-ray diffraction (XRD) analysis (Figure 3b) indicated changes in the GO precursor and the carbon-based structure after synthesis. The profile of the PcZr-RGO diffractogram differs significantly from the GO, which is dominated by a high-intensity reflection at 2Θ around 11°. The pattern of the obtained hybrid corresponds more closely to the structure of a reduced graphite oxide in the form of expanded flakes (like in the case of graphene with wide reflection around 26°) with relatively low oxygen content and a more developed specific surface area [44,45].

The composition analysis (performed using inductively coupled plasma optical emission spectrometry, ICP-OES) showed the content of zirconium equal to 280 mg/kg, which is about 0.2 wt.% of PcZr in the material.

**Figure 4:**
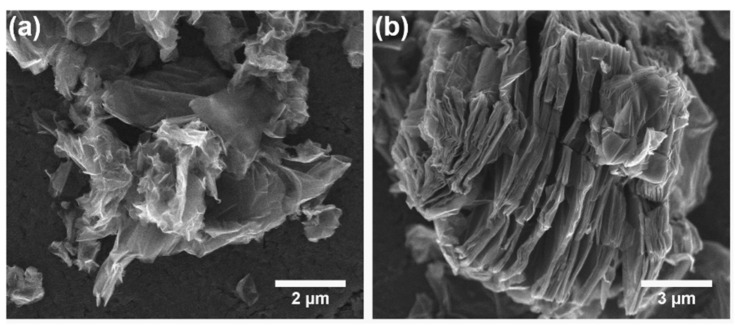
SEM images of PcZr-RGO obtained by the solvothermal method in dimethyl sulfoxide (**a**) and 1,2,4-trichlorobenzene (**b**).

Scanning electron microscopic (SEM) observations were carried out to investigate the influence of the synthesis procedure on the morphology of the product. Figure 4 shows the representative SEM images of the PcZr-RGO samples obtained by the solvothermal method in DMSO and TCB, where a complex morphology of the samples can be seen. PcZr-RGO hybrids showed a layered, wrinkled, and fluffy microstructure. The RGO flakes exhibited relatively small dimensions (from 1 to 10 μm) and separated layers, without extensive agglomeration. This phenomenon may be caused by a large amount of functional groups, such as hydroxyl and carboxyl groups, on the edges, as well as carboxyl and epoxide groups in the inner part of the particles. The distance between layers in PcZr-RGO was in the range from several dozen to several hundred nanometers (Figure 4b). Slightly bigger (up to 30 μm) plate-like particles were observed for the sample obtained using TCB. The samples were also analyzed using energy dispersive X-ray spectroscopy (EDX) to determine their chemical composition. The quantitative analysis based on the recorded spectra of two different kinds of samples (obtained in DMSO and TCB) is collected in Table 1, showing a composition of C, O, and Zr elements in PcZr-RGO.

### 2.2. Optical Measurements

Optical spectroscopy was used to describe not only the optical properties of the material under investigation but also to determine its specific surface area, ROS generation, and photocatalytic dye degradation.

#### 2.2.1. Absorption and Photoluminescence Spectra

**Figure 5:**
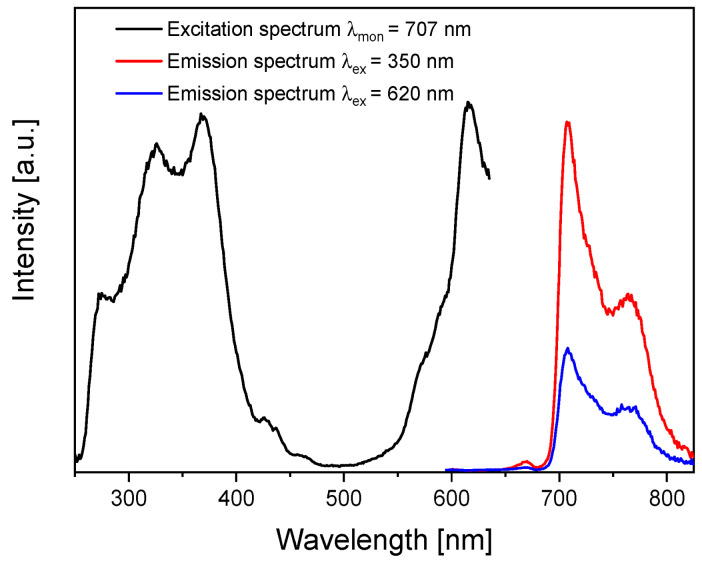
Photoluminescence excitation and photoluminescence spectra of PcZr-RGO in DMSO.

The initial optical study involved measuring the absorption spectrum (Figure 1). The band positions indicated possible ranges of excitations, which were confirmed by registering the photoluminescence excitation spectrum (Figure 5). As in the absorption spectrum, two excitation ranges in the UV (Soret, B band) and red (in Figure 5, seen as a satellite band in the Q band region at 620 nm) regions were observed when monitoring PcZr emission at 707 nm. Thus, two wavelengths, 350 nm and 620 nm, were used for photoluminescence measurements (Figure 5). Regardless of the excitation wavelength, the emission maxima in both spectra are located at 707 nm with the Stokes shift of about 22 nm.

#### 2.2.2. Surface Area Analysis

**Figure 6:**
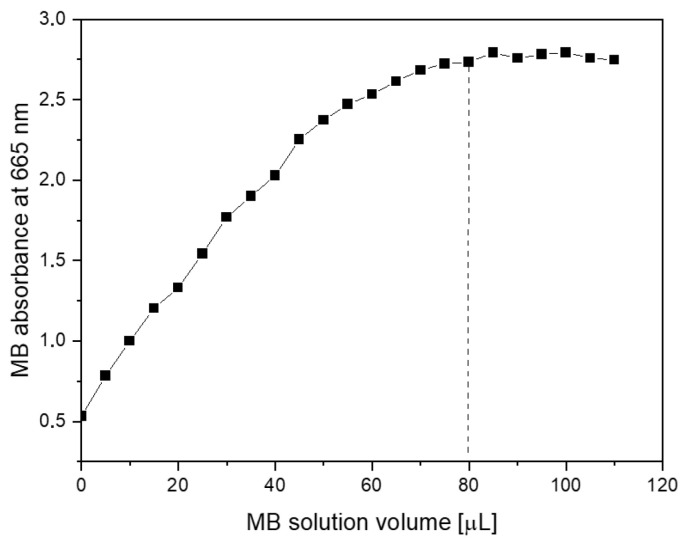
Dependence of absorption intensity in the maximum of methylene blue band on its concentration in aqueous dispersion of PcZr-RGO.

Surface area measurements were obtained by starting with an aqueous dispersion of the investigated material at a known concentration. Then, increasing amounts of the methylene blue (MB) were successively added to the suspension. The evolution of absorption spectra was recorded step by step. From the graph of the dependence of absorption intensity (MB maximum at 665 nm) on the dye concentration, one can determine the saturation point (dotted line in Figure 6). At this point, the MB fully covers the GO surface, and adding more dye causes the (R)GO-MB conjugate to precipitate. According to Montes-Navajas et al. [46], the area covered by 1 µg of MB was estimated to be 2.54 m^2^ for diluted GO suspensions. Considering this value, the surface area of the PcZr-RGO was calculated to be approximately 900 m^2^g^−1^.

#### 2.2.3. ROS Generation Abilities

**Figure 7:**
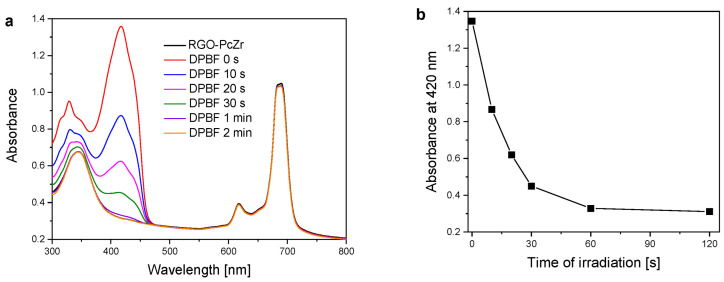
Spectra of DPBF bleaching in the presence of PcZr-RGO in DMSO under light (**a**). Dependence of DPBF maximum absorbance (at 420 nm) on time of irradiation (**b**).

Pcs, in general, are well-known photoactive compounds that can generate ROS under light illumination. 1,3-diphenylisobenzofuran, DPBF, is a dye used to estimate this activity. DPBF molecules are reacting with singlet molecular oxygen (and other ROS), which is observed as a decrease in their absorption band. Figure 7 shows the spectra of DPBF bleaching in the presence of PcZr-RGO in DMSO under red light irradiation, together with the dependence of maximum dye absorbance (at 420 nm) on the irradiation time. The DPBF absorption band intensity decreased continuously, with complete disappearance occurring after two minutes.

#### 2.2.4. Photodegradation of Model Dyes in Water

The photodegradation tests of selected model dyes in the presence of PcZr-RGO material in water were performed based on the absorption measurements. The results are summarized in Table 2. The graphs present the decrease in the dyes’ maximum absorbance over time. Selected registered spectra are presented in Figure S2 (Supplementary Materials). The black lines indicate changes observed in the darkness, most probably related to the adsorption process of the dye on the material surface. The red lines show the process of photodegradation of the dye under the influence of red light irradiation.

#### 2.2.5. Stability and Reusability of RGO-ZrPc Composite Material

In all tests performed, regardless of the dye, the absorbance decrease for RGO-PcZr after the third measurement cycle was below 1.2%. The decrease in photodegradation level varied for each dye. In the case of methylene blue, a decrease in reduction from 70% to 68% was observed after the second cycle and to 65% after the third wash. For Brilliant Green, there was virtually no decrease in the composite’s photoactivity; 87%, 86%, and 83% of the dye was photodegraded in the first, second, and third cycles, respectively. For Rhodamine B, the percentage of photodegradation in the first cycle of the reusability test was slightly higher than in the initial photodegradation studies, reaching approximately 31%. In the second cycle, this percentage decreased slightly to 29%, and in the third, to 26%. For Eriochrome Black T, also, a higher level of photodegradation was observed in the first cycle, reaching 55%, but the activity reduction was more significant, reaching 47% and 42% after the second and third cycles, respectively.

## 3. Discussion

To fabricate the PcZr-RGO hybrid, the solvothermal reaction in the presence of GO and PcZrCl_2_ was proposed. During the reaction at high pressure and elevated temperature, the flakes of GO were partially reduced to RGO. Additionally, they were also exfoliated, significantly increasing the material’s specific surface area, which was three times larger than that of unreduced GO [46]. The proposed material, where no additional ligand was used for PcZr immobilization, makes the preparation of such material simpler and less expensive in comparison to the conventional method, which involves obtaining laterally substituted Pc complexes, separating the products, and then attaching them to the GO/RGO matrix. Such a carbon-based structure has the ability to adsorb aromatic substances through the formation of van der Waals interactions.

The effectiveness of reactions performed in two different solvents, i.e., in DMSO and TCB, would indicate that the covalent and coordination attachment of zirconium phthalocyanine to the graphite oxide is ensured through the reaction with the –OH and –COOH groups rather than with the C=O. The type of solvent also influenced the morphology of the samples. Those obtained in DMSO were more exfoliated and had smaller particle sizes.

Both the ICP-OES and EDX analyses confirmed the presence of zirconium in the material. The discrepancy in the final Zr content may result from two factors. Firstly, the EDX results for low concentrations of elements are imprecise. Secondly, this method primarily records the composition of the material on the surface of the sample, where most of the PcZr is bound, resulting in a higher registered Zr content. Nevertheless, the concentration of phthalocyanine in the material is relatively high. Jurow and coworkers, who described a similar system [32], provided the C:Zr molar ratio as 31:0.006. Converted to a weight ratio, this would be approximately 1:0.001. And assuming that the gross formula of the graphite oxide we use is CO_0.5_H_0.2_, we calculated that in our case, based on the results of the ICP-OES analysis, the C:Zr weight ratio is approximately 1:0.0015, and in the case of the EDX analysis, this ratio averages 1:0.014 (deviations of significance for different detection fields vary within the range of 1:0.01–0.018). Based on the PcZr concentration and the data presented by Nyokong and Atunes [7], the quantum efficiency of ROS generation by the investigated system was higher than in the case of PcZn in DMSO, which is the reference compound for calculations. This confirms the assumption that in the hybrid material, RGO can play the role of an optical antenna and transfer the energy of absorbed photons to zirconium phthalocyanine.

The absorption and luminescence spectra of RGO-PcZr demonstrated the presence of the photosensitizer and its optical activity. The characteristic Pc bands were clearly visible in both spectra. There was also an indication of the concentration of fluorescence self-quenching. The maximum of the Soret band was expected to be around 350 nm. However, at this wavelength, there was a decrease in band intensity, reaching a minimum at 347 nm. This phenomenon has also been observed previously [40,41].

The decrease in absorption during the dark phase of the experiment with dye solutions indicated the material’s very high adsorption capacity, resulting from its developed specific surface area and affinity for aromatic substances. The chemical nature of the dyes varies, which affects their affinity for RGO. Evidently, there was a decrease in dye absorption during red light irradiation, caused by photodegradation of the pigments due to reactive oxygen species generated by phthalocyanine. The most significant discoloration occurred within the first few minutes of irradiation and continued throughout the testing period. The greatest overall decrease in absorption was observed for Brilliant Green (88%), followed by methylene blue (70%), Eriochrome Black T (50%), and Rhodamine B (25%). These values are comparable to or higher than those reported in the literature for similar photosensitive composite systems [17,33,34,35,36,37,38]. In the case of methylene blue, in various works based on materials containing Pc and GO (or RGO), or one of these components, photoreduction efficiency ranges from 40% to 99% [47]. For Brilliant Green, for the same types of materials, it ranges between 80 and 100% [37]; for Eriochrome Black T, up to 65% [38]; and for Rhodamine B, from 40% to 100% [17,48,49]. Of course, these parameters decrease with the number of dye photodegradation cycles. In our case, these parameters were lower due to the lack of an inorganic component in the system, as discussed above. We also observed a decrease in activity during tests to determine the reusability of the photocatalyst for the photoreduction of model dyes. We believe this is related to the fact that some material is lost during rinsing (washing off the catalyst).

## 4. Materials and Methods

### 4.1. Synthesis of Precursors

All reagents for zirconium complex and GO syntheses were used without further purification: ZrCl_4_ (99%, ABCR, Karlsruhe, Germany), 1,2-dicyanobenzene (>99%, Alfa-Aesar, Karlsruhe, Germany), 1-methylnaphthalene (98%, Alfa-Aesar, Karlsruhe, Germany), 1,2,4-trichlorobenzene (99%, Alfa-Aesar, Karlsruhe, Germany), DMSO (99%, Alfa-Aesar, Karlsruhe, Germany), fuming nitric acid (98–100%, Merck, Darmstadt, Germany), synthetic graphite < 20 μm (Sigma-Aldrich, Burlington, MA, USA), potassium chlorate (99%, Alfa-Aesar, Karlsruhe, Germany), and 10 M HCl (Avantor Performance Materials Poland SA, Gliwice, Poland). The modified Brodie method to synthesize highly oxidized graphite oxide was adapted from the work of Szabó and coworkers [50] and optimized as previously described [27,48]. The dichloro-zirconium phthalocyanine complex was obtained from 1,2-dicyanobenzene and ZrCl_4_ in 1,2,4-trichlorobenzene and 1-methylnaphthalene, according to a method described in detail in our previous work [40,41].

### 4.2. Solvothermal Synthesis of RGO-PcZ Hybrid

To prepare the hybrid PcZr-RGO sample, 0.5 g of graphite oxide was dispersed in 70 mL of DMSO (or 1,2,4-trichlorobenzene) for 30 min. using a 900 W, 22 kHz ultrasound disperser (UZDN M900-T, Akademprylad, Sumy, Ukraine). Then, 0.058 g (0.01 M) of PcZrCl_2_ was added to the suspension and dispersed by ultrasonication for 15 min. The reaction mixture was poured into a 100 mL Teflon-lined stainless-steel autoclave and was kept at 200 °C for 5 h in a dryer with forced air circulation. After cooling the reactor, the product was separated by ultracentrifugation, washed three times with DMSO and three times with a 1:1 cold water-ethanol mixture, and dried.

### 4.3. Analyses of Structure, Morphology, and Composition

The infrared spectroscopy analysis was performed using a NicoletTM iS50 FTIR spectrometer (Thermo Fisher Scientific Inc., Waltham, MS, USA), with a measurement range of 4000–100 cm^−1^. The samples for measurements were prepared in the form of KBr pellets or nujol mull.

Raman spectroscopy analysis was performed in the measurement range of 100–1800 cm^−1^ with μRaman inVia by Renishaw, Great Britain, supported with a CCD camera and a 514 nm laser line. The structure was analyzed with an X-ray powder diffractometer X’Pert Pro by PANalytical, Almelo, the Netherlands.

Zirconium content was determined by the ICP-OES method with 7000 iCAPICP-OES by Thermo Scientific (Waltham, MA, USA). 200 mg of vacuum-dried sample (PcZr-RGO) was burnt in a ceramic crucible with a lid (950 °C, 5 h). The ashes from the crucible and the lid were dissolved in nitric acid and analyzed. The result was calculated on an mg/kg scale with a calculation error of less than 2%.

The morphology and chemical composition of the samples were determined using a field emission scanning electron microscope (SEM, FEI Nova NanoSEM 230, FEI Company as a part of Thermo Fisher Scientific, Hillsboro, OR, USA) equipped with an energy-dispersive X-ray spectrometer (EDAX Apollo 40 SDD, AMETEK, Inc., Berwyn, PA, USA) with a resolution better than 135 eV and compatible with Genesis EDAX Microanalysis Software 6.0. The samples were prepared by dispersing materials in ethanol and depositing them on a silicon stub. SEM images were taken with an acceleration voltage of 10.0 kV in a beam deceleration mode to show the samples’ detailed features. Energy dispersive X-ray spectroscopy (EDS) analyses were also performed at 10.0 kV on a large area of the samples. Signals from three randomly selected areas were collected to ensure satisfactory statistical averaging.

### 4.4. Optical Spectroscopy Measurements

Absorption spectra were measured using a Cary 5000 UV-Vis-NIR spectrophotometer (Agilent, Santa Clara, CA, USA). Spectra were resolved in the NIR region at <0.2 nm and in the UV-Vis region at <0.05 nm. For sample characterization, 5 mg of the powder was dispersed in 5 mL of DMSO with a high-power ultrasound disperser. Measurements were taken in 10 × 10 mm quartz cuvettes with slit and step settings of 1 nm.

The spectrophotometer FLS980 (Edinburgh Instruments Ltd., Livingston Village, Great Britain) in Czerny–Turner configuration with Vis detector was used for luminescence excitation and emission spectra registration. The spectra were recorded for PcZr-RGO suspension in DMSO (0.5 mg/mL). Measurements were performed in 10 × 10 mm quartz cuvettes.

The methylene blue (Alfa Aesar, Karlsruhe, Germany) dye sorption method was used to estimate the surface area of the material [46]. The PcZr-RGO suspension (0.25 mg/mL) was titrated with a methylene blue stock solution (0.3 mg/mL), and the absorption spectrum was recorded with each subsequent addition of the MB solution.

For ROS determination, a method based on absorption measurements in the presence of a scavenger was used as described elsewhere [51]. PcZr-RGO was dispersed in 2 mL of DMSO to obtain the absorbance level of the PcZr Q band at about 1. Then, 20 µL of DPBF (1,3-diphenylisobenzofuran, Alfa-Aesar, Karlsruhe, Germany) solution in DMSO (0.1 mg/mL) was added to the cuvette with PcZr-RGO dispersion and mixed (the absorbance level of the DPBF band maximum at 410 nm was around 1). The cell was irradiated with a wide wide-spectrum red lamp (150 W red lamp, 600–950 nm range, Philips, Eindhoven, The Netherlands) for 10 s, 20 s, 30 s, 60 s, and 120 s, till the total bleaching of DPBF.

Photodegradation of organic molecules was tested on four dyes: methylene blue, Rhodamine B, Brilliant Green, and Eriochrome Black T (Alfa-Aesar, Karlsruhe, Germany), monitoring their absorption spectra. The pH of the media was around 6. Before the red light illumination (150 W red lamp, 600–950 nm range, Philips, Eindhoven, The Netherlands), the dyes were mixed in the darkness with the PcZr-RGO suspension (0.25 mg/mL) for 60 or 90 min. Tests were performed in water in 10 × 10 mm quartz cuvettes. The initial dye concentrations equaled 2 × 10^−4^ mol/L, and the illumination time was up to 180 min.

RGO-PcZr stability and potential reusability were also tested using initially the same hybrid and dye concentrations as described above. At each stage, a “zero” absorption spectrum was recorded immediately after adding the dye to the RGO-ZrPc suspension. The sample was kept 90 min. in the dark, and the absorption spectrum was measured every 30 min. The sample was then irradiated for 90 min, and absorption spectra were collected every 30 min. After the measurement, the sample was separated using an ultracentrifuge (6000 rpm) and washed a few times (3 × DMSO, 3 × ethanol:H_2_O mixture). After washing, the material was resuspended in water. This cycle was then repeated two more times for each dye.

## 5. Conclusions

This work proposed a fast and effective method of functionalizing graphite oxide with zirconium phthalocyanine. The resulting material was flakes of partially reduced GO with a PcZr content of approximately 0.2 wt.%. UV-Vis and photoluminescence spectra confirmed the presence of the Pc complex in the material, which exhibited ROS generation ability under red light irradiation. This hybrid had a high surface area of approximately 900 m^2^/g and a high affinity for aromatic and heterocyclic substances. Selected organic dye models were partially adsorbed onto the flakes and photodegraded when irradiated. The highest dye removal efficiency was achieved with Brilliant Green, reaching 88% using the selected procedure. The obtained results indicate the usefulness of the material for processes that require an oxidizing environment without introducing external oxidants and that can be exposed to light. For example, it could be used as a filter to purify polluted water and air.

## Figures and Tables

**Table 1:** Chemical composition (wt.%) calculated from EDS spectra for PcZr-RGO samples obtained with dimethyl sulfoxide or 1,2,4-trichlorobenzene.

Synthesis of PcZr-RGO	Averaged Chemical Composition (wt.%) ^(^*^)^		
	C	O	Zr
in DMSO	82.4	16.1	1.5
in TCB	73.1	19.3	1.5

^(^*^)^ The relative errors of the EDS method are less than 2%, 4%, and 50% for main (above 20 at.%), major (20–5. at.%) and trace (1–0.1) elements, respectively.

**Table 2:** Photodegradation tests of four model dyes in water in the presence of PcZr-RGO.

Model Dye and Its Structural Formula	Dependence of Dyes’ Maximum Absorbance on Time Observed in the Dark (Black Line) and Under Irradiation (Red Line)	Adsorption (A) and Photodegradation (PD) [%]
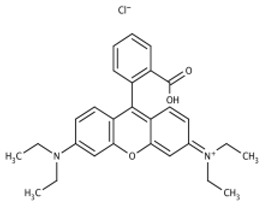 Rhodamine B	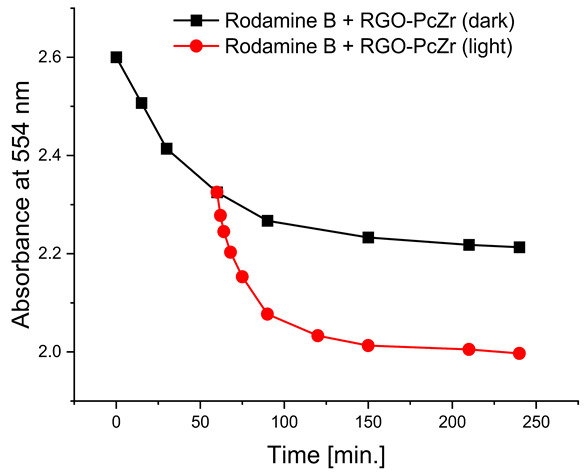	A—17% PD—25%
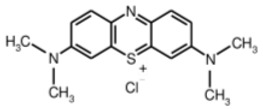 Methylene Blue	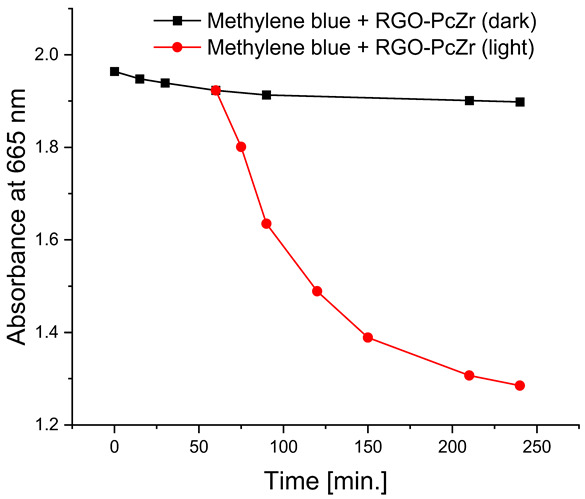	A—7% PD—70%
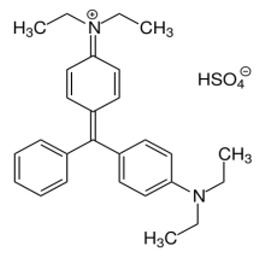 Brilliant Green	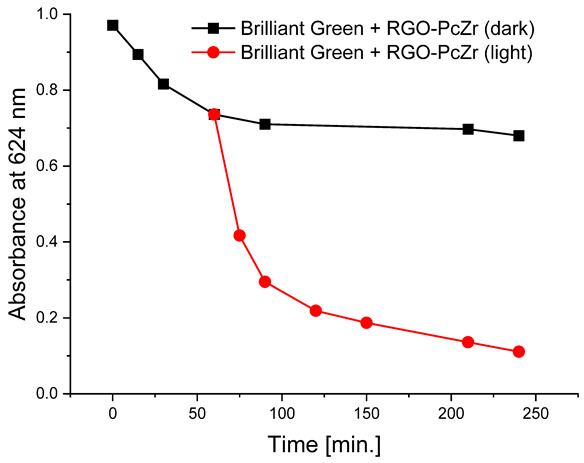	A—38% PD—88%
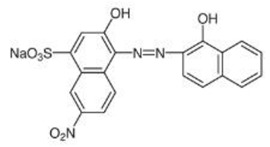 Eriochrome Black T	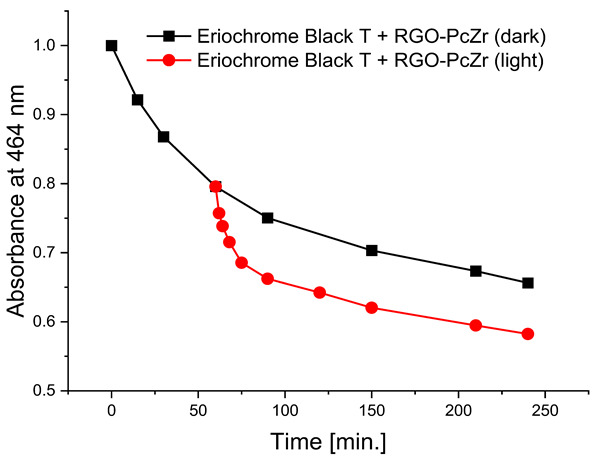	A—44% PD—50%

## Data Availability

The original contributions presented in this study are included in the article/Supplementary Materials. Further inquiries can be directed to the corresponding author.

## Supplementary Materials

The following supporting information can be downloaded at: https://www.mdpi.com/article/10.3390/molecules30214242/s1, Figure S1: Absorption spectra of PcZr-RGO obtained by solvothermal reaction in dimethyl sulfoxide and in 1,2,4-trichlorobenzene; Figure S2: Absorption spectra of organic dyes in water exposed to PcZr-RGO and light; Description of Control test of PcZrCl_2_ adsorption on RGO surface; Figure S3: Far region infrared spectra of PsZrCl_2_, RGO and RGO-ZrPc composite material; Figure S4. Middle region infrared spectra of PsZrCl_2_, RGO and RGO-ZrPc composite material. Figure S5. Absorption spectra of solvothermally reduced RGO suspension, PcZrCl_2_ solution, RGO/PcZrCl_2_ mixture (mixed and after 5 h), washed and redispersed precipitate, PcZr-RGO composite material suspension in DMSO.
