# Supplementary material for: Synthesis, Structure, and Properties of Reduced Graphite Oxide Modified with Zirconium Phthalocyanine as a Catalyst for Photooxidation and Dye Photodegradation
Source: Molecules. 2025 Oct 31;30(21):4242. doi: 10.3390/molecules30214242 (PMC12608119; doi:10.3390/molecules30214242)
Supplement: Supplementary file 1 [file molecules-30-04242-s001.zip › molecules-3885137-supplementary.pdf]

Synthesis, structure and properties of reduced graphite oxide modified with zirconium phthalocyanine as catalyst for photooxidation and dyes photodegradation

Y. Gerasymchuk[1], A. Wędzyńska[1], D. Szymański[1], V. Chernii[2], I. Tretyakova[2], A. Lukowiak[1*]

Supplementary Materials

Reaction efficiency of $PcZrCl_2$ with GO under solvothermal condition depends on the solvent used in the process. For the chosen system, better results were obtained after reaction in N,N'-dimethylsulphoxide (DMSO) than in trichlorobenzene (TCB) as confirmed by the absorption spectra of powders redispersed in DMSO after synthesis (Fig. S1).

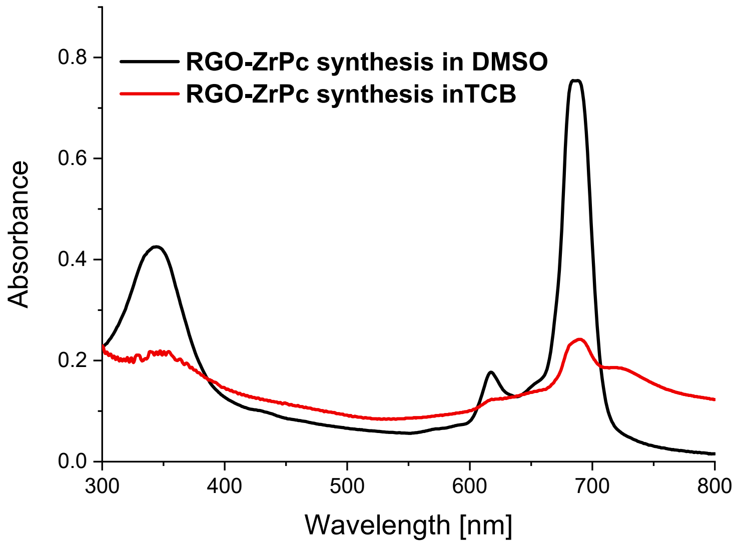

Figure S1. Absorption spectra of PcZr-RGO obtained by solvothermal reaction in dimethyl sulfoxide and in trichlorobenzene.

The infrared spectroscopy analysis (FIR 100-400, MIR 400-4000) was performed using Biorad 575C spectrometer, with measurement range of 4000–30 cm^{-1}. The samples were prepared in the form of KBr pellets for measurements. The mid- and far-infrared spectroscopic measurement (FTIR) data for the obtained PcZr-RGO material are presented below:

$PcZrCl_2$: 1685 (w), 1605 (w), 1500 (m), 1465 (w), 1415 (m), 1385 (w), 1330 (s), 1310 (w), 1285 (s), 1155 (m), 1115 (s), 1070 (s), 1050 (s), 950 (w), 890 (s), 870 (w), 825 (m), 790 (m), 765 (m), 745 (s), 730 (s), 630 (w), 565 (w), 500 (m), 430 (m), 345 (m) [m_{asym}(Zr–Cl)], 315 (s) [m_{sym}(Zr–Cl)].

GO: C=O 1720-1700 cm^{-1}, υ (C–O) – 1220-1200 cm^{-1}, υ (O–H) – 3600-3550 cm^{-1}.

For the PcZr-RGO, a disappearance of signals attributed to the different types of Zr–Cl bond vibrations and the appearance of corresponding signals of Zr–O bond vibration in the range of 400-750 cm^{-1} were observed. Also, most of the signals corresponding to the vibration bonds in phthalocyanine macrocycle were also observed in the hybrid but with much lower intensity due to the very low concentration of PcZr.

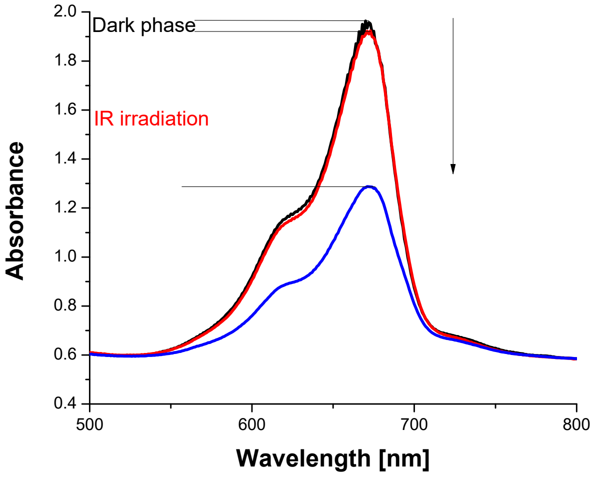

Methylene Blue

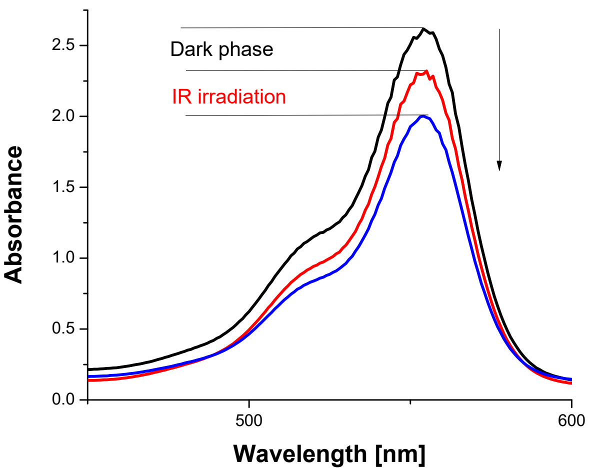

Rhodamine

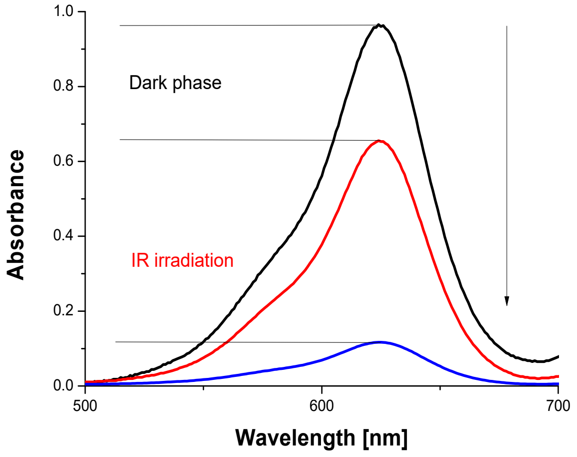

Brilliant Green

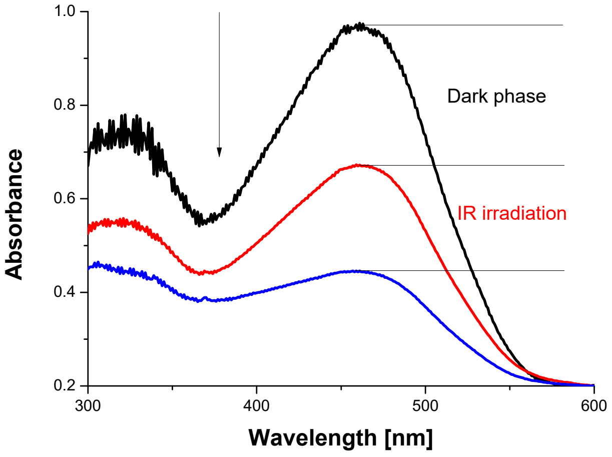

Eriochrome Black T

Figure S2. Absorption spectra of organic dyes in water exposed to PcZr-RGO and light. Black lines – initial dyes spectra in the presence of PcZr-RGO dispersion; red curves – spectra after experiment performed in darkness; blue lines – spectra after experiment performed under light irradiation.

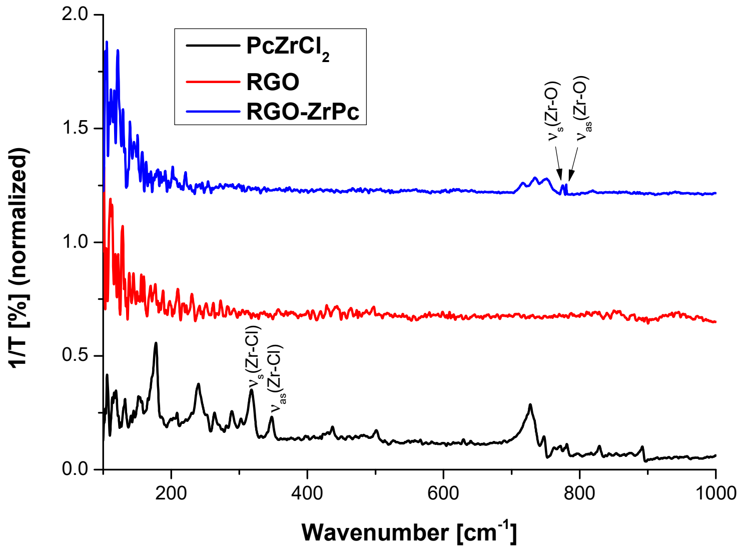

Figure S3. Far region infrared spectra of PsZrCl2, RGO and RGO-ZrPc composite material.

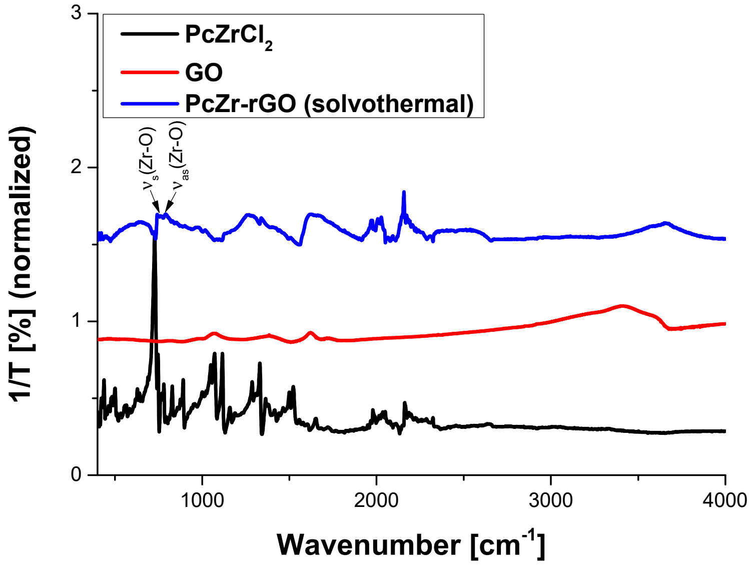

Figure S4. Middle region infrared spectra of PsZrCl2, RGO and RGO-ZrPc composite material.

A suspension of RGO (1 mg/ml, prepared by ultrasonification) and a solution of PcZrCl2 (0.1 mg/ml) in DMSO were prepared. Absorption spectra were recorded for both components. Absorption spectra were also recorded immediately after mixing the two components, and 5 hours after mixing. The product was precipitated with water and the resulting precipitate was then separated using an ultracentrifuge (12,000 rpm) and washed according to the standard procedure used for washing the composite material after solvothermal synthesis (washing with 2xDMSO, 2xEtOH, 1xH_2O). After drying, the material was redispersed by ultrasonification in DMSO, and an absorption spectrum was recorded for it. The results are presented in Fig. S5. For comparison, the spectrum of the composite material obtained by the solvothermal method was also included.

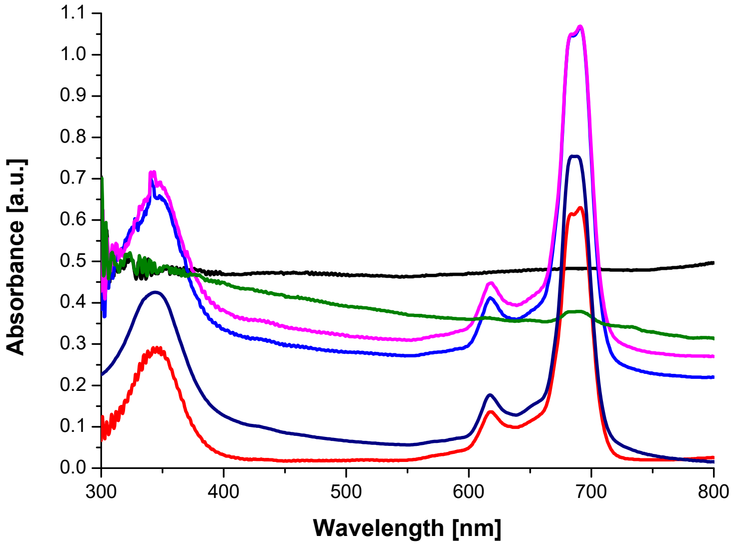

Figure. S5 Absorption spectra of solvotermally reduced RGO suspension - (black line), PcZrCl2 solution - (red line), RGO/ PcZrCl2 mixture (directly after mixing) - (blue line), RGO/ PcZrCl2 mixture (after 5h) - (pink line), washed and redispersed precipitate - (green line), RGO-ZrPc composite material suspension in DMSO - (dark blue line).

The obtained absorption test results clearly indicate the instability of the material obtained by adsorption of zirconium phthalocyanine onto reduced graphite oxide. With standard rinsing procedures, the phthalocyanine complex washes out of the material, remaining only in trace amounts.
